# Urinary biomarkers predict advanced acute kidney injury after cardiovascular surgery

**DOI:** 10.1186/s13054-018-2035-8

**Published:** 2018-04-26

**Authors:** Jian-Jhong Wang, Nai-Hsin Chi, Tao-Min Huang, Rory Connolly, Liang Wen Chen, Shih-Chieh Jeff Chueh, Wei-Chih Kan, Chih-Cheng Lai, Vin-Cent Wu, Ji-Tseng Fang, Tzong-Shinn Chu, Kwan-Dun Wu

**Affiliations:** 1 Division of Nephrology, Department of Internal Medicine, Chi Mei Medical Center, Liouying, Tainan, Taiwan; 2 NSARF group (National Taiwan University Hospital Study Group of ARF), Taipei, Taiwan; 3 Department of Surgery, National Taiwan University Hospital, Taipei, Taiwan; 4 Division of Nephrology, Department of Internal Medicine, National Taiwan University Hospital, Taipei, Taiwan; 5 School of Biotechnology, Dublin City University, Glasnevin, Dublin 9, Ireland; 6 Cleveland Clinic Lerner College of Medicine and Glickman Urological and Kidney Institute, Cleveland Clinic, Cleveland, USA; 7 Division of Nephrology, Department of Internal Medicine, Chi-Mei Medical Center, Tainan, Taiwan; 8 Department of Intensive Care Medicine, Chi Mei Medical Center, Liouying, Tainan, Taiwan; 9 Chang Gung University College of Medicine, Taoyuan, Taiwan; 10 Kidney Research Center, Department of Nephrology, Chang Gung Memorial Hospital, Taoyuan City, Taiwan

**Keywords:** Biomarkers, Acute kidney injury, Hemojuvelin, Kidney injury molecule-1, Neutrophil gelatinase-associated lipocalin, α-Glutathione S-transferase, π-Glutathione S-transferase, Liano’s score

## Abstract

**Background:**

Acute kidney injury (AKI) after cardiovascular surgery is a serious complication. Little is known about the ability of novel biomarkers in combination with clinical risk scores for prediction of advanced AKI.

**Methods:**

In this prospectively conducted multicenter study, urine samples were collected from 149 adults at 0, 3, 6, 12 and 24 h after cardiovascular surgery. We measured urinary hemojuvelin (uHJV), kidney injury molecule-1 (uKIM-1), neutrophil gelatinase-associated lipocalin (uNGAL), α-glutathione S-transferase (uα-GST) and π-glutathione S-transferase (uπ-GST). The primary outcome was advanced AKI, under the definition of Kidney Disease: Improving Global Outcomes (KDIGO) stage 2, 3 and composite outcomes were KDIGO stage 2, 3 or 90-day mortality after hospital discharge.

**Results:**

Patients with advanced AKI had significantly higher levels of uHJV and uKIM-1 at 3, 6 and 12 h after surgery. When normalized by urinary creatinine level, uKIM-1 in combination with uHJV at 3 h post-surgery had a high predictive ability for advanced AKI and composite outcome (AUC = 0.898 and 0.905, respectively). The combination of this biomarker panel (normalized uKIM-1, uHJV at 3 h post-operation) and Liano’s score was superior in predicting advanced AKI (AUC = 0.931, category-free net reclassification improvement of 1.149, and *p* <  0.001).

**Conclusions:**

When added to Liano’s score, normalized uHJV and uKIM-1 levels at 3 h after cardiovascular surgery enhanced the identification of patients at higher risk of progression to advanced AKI and composite outcomes.

**Electronic supplementary material:**

The online version of this article (10.1186/s13054-018-2035-8) contains supplementary material, which is available to authorized users.

## Background

Patients with acute kidney injury (AKI) have increased mortality, prolonged hospital stay and accelerated progression to chronic kidney disease (CKD) [1]. AKI is associated with high morbidity and mortality despite advances in modern medical care [2, 3]. Early and exact detection of potentially significant AKI is important in clinical practice and could lead to timely management [4]. Serum creatinine has traditionally served as a surrogate of renal function, despite its limitations as a diagnostic predictor of AKI [5]. The limitations of serum creatinine include a lack of steady-state conditions in critically ill patients, with the determinants of serum creatinine (rate of production, apparent volume of distribution and rate of elimination) being variable in the ICU setting.

Novel biomarkers can detect renal tubular injury earlier than serum creatinine in the setting of AKI [6–8]. As these markers correlate with renal tubular injury or function, their concentrations in urine over time, alone or in combination, could provide important information on the progression of AKI [9]. However, most of those patients who develop AKI experience a milder form of AKI (e.g., Kidney Disease: Improving Global Outcomes (KDIGO) stage 1) with transient shifts in serum creatinine, and do not progress to more advanced stages of AKI (KDIGO stage 2 or 3) or require acute dialysis [10–12]. Whereas previous studies have attempted to predict AKI (defined as worsening of KDIGO stage) rather than immediate assessment after potential kidney insult, a panel of biomarkers could be used to predict progression of AKI to advanced AKI or mortality among patients after kidney injury (e.g., cardiovascular surgery) [10, 13, 14]. Therefore, we validated three urinary markers extensively upregulated in renal proximal tubules in response to ischemia-reperfusion AKI: urinary hemojuvelin (uHJV) [8], urinary kidney injury molecule-1 (uKIM-1) [15] and urinary α-glutathione S-transferase (uα-GST) [16], and one marker of distal tubule damage, urinary π-glutathione S-transferase (uπ-GST) [16] (see Additional file 1). We further measured urinary neutrophil gelatinase-associated lipocalin (uNGAL), the well-studied and established inflammatory biomarker identified both in proximal and distal renal tubular damage [17], to validate the prediction of AKI following sequential biomarker measurements after cardiovascular surgery.

Moreover, multiple AKI severity scores have also been derived to predict patient outcome [18]. We further compared the contribution of the clinical models of Liano’s score (see Additional file 1) [19], Cleveland Clinic acute renal failure score [20], and Sequential Organ Failure Assessment (SOFA) score [21], and combined these with the predictive power of the urinary biomarkers in an effort to identify and validate the best prediction model for advanced AKI and composite outcomes in patients undergoing cardiovascular surgery.

We hypothesized that some combinations of clinical scores and these site-orientated renal biomarkers would provide better diagnostic accuracy than either alone. In addition, these combinations might involve biomarkers that reflect differing aspects of the pathogenesis of advanced AKI in this population.

## Methods

### Study population

This study was conducted by the biomarker investigation group from the National Taiwan University Study Group on Acute Renal Failure (NSARF) (see Additional file 1), using a multicenter, prospectively constructed database of AKI [22–26]. Patients undergoing cardiovascular surgery (including coronary bypass, valvular operations and aortic aneurysm repair) between August 2009 and December 2014, were enrolled prospectively from a tertiary center in northern Taiwan and two regional hospitals in central and northern Taiwan. Patients with the following conditions were excluded: those who were younger than 18 years of age, those who were diagnosed with AKI under the KDIGO definitions during index hospitalization before surgery, those who had undergone renal replacement therapy, those who had a history of nephrectomy or renal transplantation and estimated glomerular filtration rate (eGFR) < 30 mL/1.73 m^2^ at the time of ICU enrollment. The enrollee was required to have a baseline serum creatinine measurement, defined as 7–180 days prior to the index hospital admission.

### Ethics and consent

This study was approved by the research ethics review board of National Taiwan University Hospital (201105040RC) along with established written informed consent. This research was carried out in accordance with the approved guidelines. Written informed consent was obtained from all participants before inclusion. This study was conducted in accordance with the Declaration of Helsinki.

### Clinical data collection

Medical records of study participants were prospectively reviewed to retrieve hospitalization data, including baseline demographic characteristics (Table 1), intervention procedures, and comorbidity status. Creatinine level with the accompanying eGFR (through the Modification of Diet in Renal Disease (MDRD) Study equation formula) was used for eGFR in this study. Among adults, the MDRD Study equation provides a clinically useful estimate of GFR in patients with stable kidney status [27]; urine output was recorded at each time point after surgery as detailed in the study protocol. Postoperative inotropic agent use was quantified as per inotropic equivalents (see Additional file 1) [23, 25, 28]. Surgery-related parameters included surgical methods, aortic clamping time and cardiopulmonary bypass time. Lengths of total admission and ICU admission were also recorded. Disease severity was evaluated as post-surgical SOFA score. The clinical classifications of Liano’s score [19] and Cleveland Clinic Foundation Acute Renal Failure Scoring System (CCF ARF score) [20] were also assessed to examine the risk of postoperative renal failure. Clinical and demographic characteristics of these patients were collected at each site on a case report form, which was sent to the coordinating center for entry into the NSARF database [29–31].

**Table 1:** Summary of baseline and clinical characteristics of the study patients

	All	No AKI or stage 1 AKI	Stage 2 or 3 AKI	*p* value
	(*n* = 149)	(*n* = 131)	(*n* = 18)	
Patient characteristics				
Age	62.36 ± 13.64	62.32 ± 13.62	62.72 ± 14.23	0.907
Gender (male)	104 (69.8%)	95 (72.5%)	9 (50.0%)	0.051
BMI	24.87 ± 3.73	24.82 ± 3.48	25.26 ± 5.33	0.735
Comorbidities				
Hypertension	77 (51.7%)	69 (52.7%)	8 (44.4%)	0.512
Diabetes mellitus	36 (24.2%)	32 (24.4%)	4 (22.2%)	0.838
COPD	4 (2.7%)	4 (3.1%)	0 (0.0%)	0.452
Liver cirrhosis	4 (2.7%)	4 (3.1%)	0 (0.0%)	0.452
Congestive heart failure	14 (9.4%)	14 (10.7%)	0 (0.0%)	0.145
Malignancy	5 (3.4%)	4 (3.1%)	1 (5.6%)	0.58
Laboratory data at admission				
Preoperative creatinine (mg/dL)	1.18 ± 0.33	1.17 ± 0.31	1.26 ± 0.43	0.388
eGFR (MDRD) (mL/min/1.73 m^2^)	63.27 ± 20.59	63.86 ± 20.29	58.97 ± 22.85	0.346
eGFR between 30 and 60 mL/min/1.73 m^2^	63 (42.3%)	56 (42.7%)	7 (38.9%)	0.756
Hemoglobin (g/dL)	13.02 ± 2.02	13.18 ± 1.96	11.83 ± 2.17	0.007*
Albumin (g/dL)	4.15 ± 0.63	4.26 ± 0.47	3.40 ± 1.03	0.003*
LVEF < 55%	35 (23.5%)	31 (23.7%)	4 (22.2%)	0.892
LVEF < 35%	10 (6.7%)	9 (6.9%)	1 (5.6%)	0.834
Perioperative condition				
Inotropic equivalents	5.83 ± 6.61	4.82 ± 4.45	13.10 ± 12.81	0.014*
Presence of CPB	93 (62.4%)	81 (61.8%)	12 (66.7%)	0.691
CPB time (min)	106.57 ± 98.87	102.23 ± 97.43	143.27 ± 111.96	0.200
Presence of cross clamp	72 (48.3%)	63 (48.1%)	9 (50.0%)	0.879
Clamp time (min)	57.41 ± 65.11	55.66 ± 65.18	78.20 ± 63.82	0.295
Operative method				
CABG	77 (51.7%)	70 (53.4%)	7 (38.9%)	0.247
Valve	64 (43.0%)	55 (42.0%)	9 (50.0%)	0.519
Aorta	20 (13.4%)	16 (12.2%)	4 (22.2%)	0.243
Post-surgery				
SOFA score	6.14 ± 2.96	5.84 ± 2.69	8.47 ± 3.92	0.015*
Respiratory	0.67 ± 0.75	0.66 ± 0.73	0.77 ± 1.01	0.608
Coagulation	0.43 ± 0.60	0.40 ± 0.57	0.69 ± 0.85	0.093
Liver	0.87 ± 0.65	0.85 ± 0.64	1.00 ± 0.71	0.432
Cardiovascular	0.28 ± 0.55	0.24 ± 0.48	0.62 ± 0.96	0.191
Central nervous system	3.08 ± 1.20	3.08 ± 1.21	3.08 ± 1.12	0.997
Renal function	0.72 ± 0.79	0.58 ± 0.66	2.07 ± 0.64	< 0.001*
Cleveland score	3.80 ± 1.62	3.79 ± 1.56	3.89 ± 2.03	0.802
Liano’s score	−0.60 ± 0.84	−0.61 ± 0.84	−0.47 ± 0.87	0.506
Length of admission (days)	18.69 ± 37.62	17.56 ± 38.51	30.75 ± 24.41	0.247
Length of ICU admission (days)	3.55 ± 3.30	3.43 ± 2.84	7.64 ± 5.35	0.027*
90-day mortality	16 (10.7%)	6 (4.6%)	10 (55.6%)	< 0.001*

Values are mean ± SD or number (percentage)

*AKI* acute kidney injury, *BMI* body mass index, *CABG* coronary artery bypass graft, *COPD* chronic obstructive pulmonary disease, *CPB* cardiopulmonary bypass, *eGFR* estimated glomerular filtration rate, *ICU* intensive care unit, *LVEF* left ventricular ejection fraction, *MDRD* Modification of Diet in Renal Disease Study equation, *SOFA score* Sequential Organ Failure Assessment score

*p < 0.05

### Sample collection

Fresh urine samples were obtained in the ICU at 0, 3, 6, 12 and 24 h after completion of surgery. Other laboratory examinations were performed as indicated clinically. Urine specimens were collected by a standardized procedure, centrifuged within 1 h and the sediments discarded. The urine samples, collected in separate polypropylene tubes containing sodium azide, were stored at − 80 °C until required. Each specimen was centrifuged (800 g at 4 °C for 5 min) and the supernatant was collected for ELISA.

### Biomarker Measurements

The urinary HJV, KIM-1 and NGAL levels were measured by a human hemojuvelin ELISA kit (USCN Life Science, Inc., Wuhan, China), human urinary TIM-1/KIM-1/HAVCR Quantikine ELISA kit (R&D Systems, USA) and a human lipocalin-2/NGAL ELISA kit (R&D Systems), respectively. All of the results were expressed in nanograms per milliliter. The lower limit of detection for HJV, KIM-1 and NGAL was 0.156, 0.046 and 0.2 ng/mL, respectively. The urinary α-GST and π-GST levels were determined using Human Alpha and Pi GST EIA Test Kits (EKF Diagnostics). The results were expressed in micrograms per liter. The inter-assay and intra-assay coefficient of variation for α-GST was 6.3% and 2.7%, respectively, and for π-GST, it was 8.6% and 3.1%, respectively. The lower limit of detection for α-GST and for π-GST was 0.3 μg/L. Assays were completed as described by the manufacturer’s protocol, and each measurement was performed in duplicate. Urinary creatinine levels were measured using the Jaffe assay, with standardization to isotope dilution mass spectrometry (IDMS)-traceable reference. Technicians performing the biomarker measurements were blinded to each patient’s clinical information. All biomarkers were measured from frozen aliquots that did not undergo any additional freeze–thaw cycles.

### Outcome definitions

The clinical primary endpoint was defined as the development of advanced AKI (stage 2 or 3, specified by the KDIGO criteria [32]), with both urine and creatinine criteria applied. The secondary outcomes were 90-day mortality after hospital discharge and composite outcomes defined as 90-day mortality after hospital discharge or advanced AKI, whichever occurred earlier.

### Statistical analysis

All analyses were performed using SPSS software, version 20 (IBM, Armonk, NY, USA), R software, version 3.2.2 (Free Software Foundation, Inc., Boston, MA, USA), and MedCalc Statistical Software, version 15.11.3 (MedCalc Software bvba, Ostend, Belgium; https://www.medcalc.org; 2015). The two-sample *t* test or Mann–Whitney rank sum test was used as appropriate to compare continuous variables; for categorical variables, the chi-square (χ2) or Fisher’s exact test was applied. Friedman two-way analysis of variance (ANOVA) was used to assess the overall difference in HJV, KIM-1, NGAL, α-GST and π-GST between the “no AKI or stage 1 AKI” and the “stage 2 or 3 AKI” groups at 0, 3, 6, 12 and 24 h after cardiovascular surgery [33]. We normalized biomarker levels with urine creatinine concentrations and analyzed them at each time point [34]. We fitted logistic regression models to examine the association between each marker (urinary NGAL, HJV, KIM-1, α-GST and π-GST) and advanced AKI, and generated an area under the receiver-operating characteristic (ROC) curve to assess the predictive accuracy of each marker. Power analysis was conducted based on the prior knowledge that the ratio of cases to controls was 4:1. We required 105 patients (21 with severe AKI and 84 without severe AKI) to achieve power of 0.8, with type I error of 0.05. This was based on the preliminary knowledge that the discriminatory power of urinary HJV to predict severe AKI was 0.7 [35]. We used a generalized additive model (GAM) (with spline) incorporating the subject-specific (longitudinal) random effects and adjusted for other clinical parameters to predict the outcomes [36, 37]. Simple and multiple generalized additive models (GAMs) were fitted to detect nonlinear effects of continuous covariates and identify appropriate cutoff point(s) for discretizing continuous covariates, if necessary, during the stepwise variable selection procedure. We defined the optimal cutoff value as odd equals to zero [38].

To assess the additive prediction ability of each biomarker compared with traditional clinical predictors, we identified clinical risk prediction models (including Liano’s score, CCF ARF score and SOFA score) that were based on area under the curve (AUC) analysis and then added each biomarker individually to this clinical model. We estimated the risk score function using a logistic regression model including clinical and biomarker variables as covariates. The Hosmer-Lemeshow logistic regression model test for goodness of fit was used to assess the calibration between the current model and the expected model. Finally, we calculated the net reclassification improvement (NRI) and integrated discrimination improvement (IDI) to estimate overall improvement in reclassification with urinary biomarkers adding to clinical variables [39]. We reclassified the patients who developed advanced AKI using 0–12%, 12–30% and > 30% for the risk categories. A *p* value <0.05 was considered significant.

Hierarchical clustering analysis was performed using the cluster program and the results were visualized using the Treeview program [40]. The biomarkers were arranged in such a way that the most similar expression profiles were placed next to each other. In the color scheme, strong positive staining is indicated as a red cube, weak positive staining as a black cube and negative staining as a green cube. Absence of staining data is indicated with a grey cube.

## Results

### Clinical characteristics

A total of 149 patients who underwent cardiovascular surgery were enrolled in this study, with subsequent time-varying sample collection. There were 46 patients who had AKI episodes as defined by KDIGO criteria (30.9%), with 28 (18.8%) having stage 1 AKI, 11 (7.4%) stage 2 AKI, and 7 (4.7%) progressing to stage 3 AKI: 6 patients progressed to dialysis-dependent AKI in the advanced AKI group. The 90-day mortality rate in this cohort was 10.7%. The clinical characteristics of patients with and without advanced AKI are described in Table 1. Patients who developed advanced AKI had lower hemoglobin and albumin levels, were administered higher inotropic equivalents and had higher SOFA scores. However, there were no statistical differences between patients with and without advanced AKI with respect to age, gender, body mass index (BMI), comorbidities, baseline kidney function, baseline chronic kidney disease (eGFR between 30 and 60 ml/min/1.73 m^2^) status and the Liano and CCF ARF disease severity scores. Clinical outcomes were worse for patients in the advanced AKI group when they had longer ICU stays and a higher 90-day mortality rate, even after discharge.

### Relationship between biomarker levels and advanced AKI

**Fig. 1:**
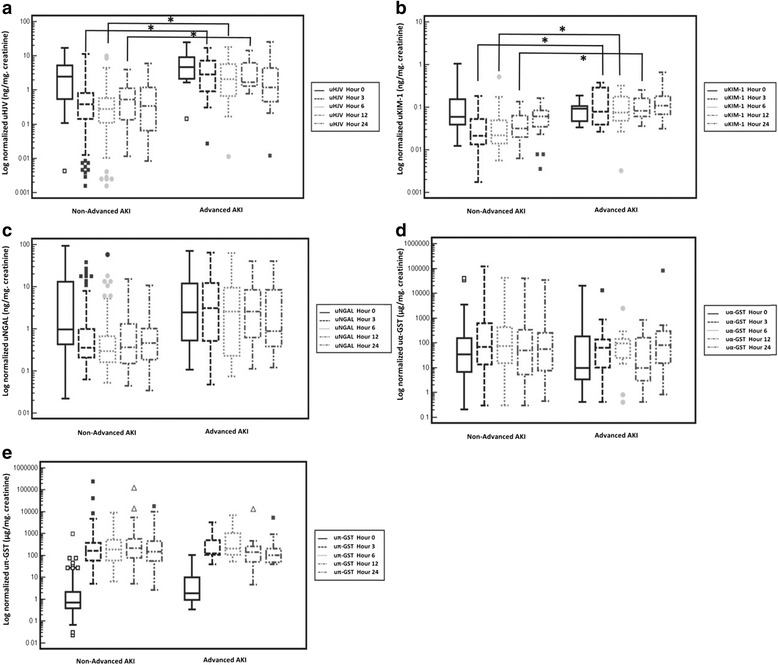
Urinary levels of five biomarkers after cardiovascular surgery. The vertical box represents the 25th percentile (bottom line), median (middle line) and 75th percentile (top line) values, whereas the vertical bars represent the intervals between maximum and minimum values. **a** Creatinine-normalized urinary hemojuvelin (uHJV). **b** Creatinine-normalized urinary kidney injury molecule-1 (uKIM-1). **c** Creatinine-normalized urinary neutrophil gelatinase-associated lipocalin (uNGAL). **d** Creatinine-normalized urinary α-glutathione S-transferase (uα-GST). **e** Creatinine-normalized urinary π-glutathione S-transferase (uπ-GST). *p < 0.05. AKI, acute kidney injury

We compared urinary biomarker levels between patients with and without advanced AKI (patients without AKI or patients with stage 1 AKI): the normalized urine biomarker data were transformed logarithmically to avoid the interference of numbers in the extreme and to approximate normal distribution in the analyses (Fig. 1). At 3 h post-surgery, normalized uHJV (*p* = 0.006), and uKIM-1 (*p* = 0.019) levels in the patients with advanced AKI were significantly higher than those without advanced AKI. Moreover, considerably higher levels of uHJV and uKIM-1 were observed at 6 and 12 h post-surgery. However, there were no significant differences in the uNGAL, uα-GST and uπ-GST levels between the patients with and without advanced AKI at these sequential time points. All biomarkers analyzed by Friedman two-way ANOVA (HJV, KIM-1, NGAL, α-GST and π-GST) were significantly different (all *p* <  0.05) between the no AKI/stage 1 AKI and the stage 2/3 AKI groups at each of the time points of 0, 3, 6, 12 and 24 h after cardiovascular surgery.

### Urinary biomarkers and the prediction of advanced AKI

**Fig. 2:**
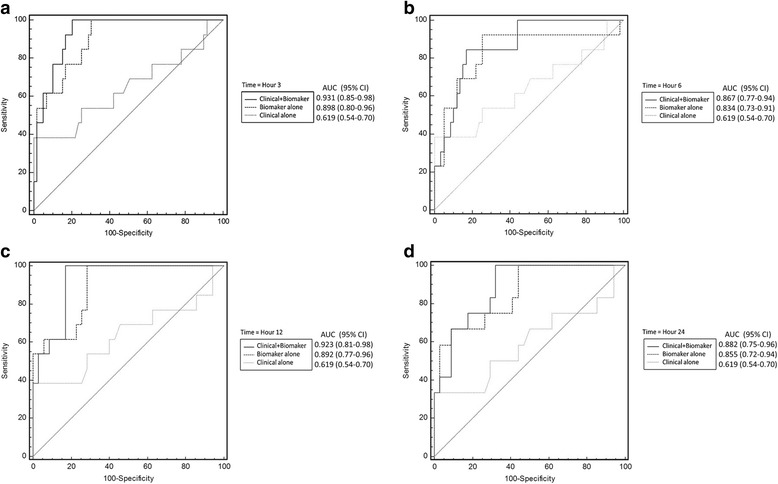
Receiver-operating characteristic (ROC) curves for the best prediction model for advanced acute kidney injury. The best biomarker panel combination alone (thick dashed line), clinical risk prediction model alone (thin dashed line) and combination of the clinical risk prediction model and combined biomarker panel (solid line) are shown at hour 3 (**a**), hour 6 (**b**), hour 12 (**c**) and hour 24 (**d**). The clinical risk prediction model is calculated from Liano’s score. The area under the ROC curve (AUC) values and 95% confidence intervals (CIs) are also shown

Among the patients who subsequently developed advanced AKI, the, uHJV biomarker had the highest AUC values, particularly at 12 h post-surgery. Following normalization with urinary creatinine concentration, the predictive ability of several urinary biomarkers was improved, especially normalized uHJV at 12 h post-surgery (Table 2). When compared to serum creatinine, normalized uHJV and uKIM-1 had better performance for predicting advanced AKI at 3 and 6 h post-surgery (Additional file 1: Table S1 and Fig. 1). The combined panel of normalized uHJV and uKIM-1 at 3 h post-surgery had the largest AUC (AUC = 0.898, 95% CI 0.80–0.96) compared to any of the other two or three biomarker combinations (Table 3 and Fig. 2). This combination had a positive predictive value of 42%, negative predictive value of 100%, sensitivity of 100% and specificity of 70%. The GAM plot was generated thereafter and demonstrated positive correlation between increased normalized uHJV and uKIM-1 at 3 h post-surgery and the risk of developing advanced AKI. The cutoff value 2.346 ng/mL for normalized uHJV and 0.047 ng/mL for normalized uKIM-1 performed best to predict advanced AKI (Additional file 1: Figure S2).

**Table 2:** Area under the receiver-operating characteristic curve at each time point for urinary biomarkers with and without normalization to urinary creatinine for predicting advanced acute kidney injury

Urinary Biomarkers	Time after enrollment	AUC	95% CI	Urinary biomarkers	Time after enrollment	AUC	95% CI
uHJV	Hour 3	0.793	0.709 to 0.862	Normalized uHJV	Hour 3	0.833	0.753 to 0.895
	Hour 6	0.802	0.720 to 0.869		Hour 6	0.808	0.726 to 0.874
	Hour 12	0.813	0.683 to 0.907		Hour 12	0.841	0.715 to 0.927
	Hour 24	0.687	0.542 to 0.809		Hour 24	0.719	0.575 to 0.835
uKIM-1	Hour 3	0.670	0.549 to 0.776	Normalized uKIM-1	Hour 3	0.819	0.710 to 0.900
	Hour 6	0.664	0.543 to 0.771		Hour 6	0.787	0.675 to 0.875
	Hour 12	0.602	0.451 to 0.741		Hour 12	0.831	0.695 to 0.923
	Hour 24	0.544	0.391 to 0.692		Hour 24	0.772	0.625 to 0.882
uNGAL	Hour 3	0.711	0.621 to 0.790	Normalized uNGAL	Hour 3	0.707	0.617 to 0.787
	Hour 6	0.754	0.667 to 0.828		Hour 6	0.691	0.600 to 0.772
	Hour 12	0.660	0.517 to 0.785		Hour 12	0.745	0.607 to 0.855
	Hour 24	0.640	0.493 to 0.769		Hour 24	0.691	0.546 to 0.813
uα-GST	Hour 3	0.504	0.419 to 0.589	Normalized uα-GST	Hour 3	0.556	0.470 to 0.639
	Hour 6	0.523	0.437 to 0.608		Hour 6	0.54	0.454 to 0.624
	Hour 12	0.573	0.487 to 0.656		Hour 12	0.61	0.524 to 0.691
	Hour 24	0.633	0.547 to 0.713		Hour 24	0.542	0.456 to 0.627
uπ-GST	Hour 3	0.669	0.584 to 0.745	Normalized uπ-GST	Hour 3	0.547	0.462 to 0.631
	Hour 6	0.779	0.702 to 0.845		Hour 6	0.591	0.505 to 0.673
	Hour 12	0.586	0.500 to 0.668		Hour 12	0.594	0.508 to 0.676
	Hour 24	0.631	0.545 to 0.710		Hour 24	0.548	0.462 to 0.632

*AUC* area under the receiver-operating characteristic curve, *CI* confidence interval, *uHJV* urinary hemojuvelin, *uKIM-1* urinary kidney injury molecule-1, *uNGAL* urinary neutrophil gelatinase-associated lipocalin, *uα-GST* urinary α-glutathione S-transferases, *uπ-GST* urinary π-glutathione S-transferases

**Table 3:** Logistic regression analysis with variables available for predicting advanced acute kidney injury and model accuracy after combining urinary biomarkers

Model	AUC (95% CI)	*p* ^b^
Hour 3		
Normalized uHJV	0.833 (0.753 to 0.895)	NA
Normalized (uHJV + uKIM-1) ^a,c^	0.898 (0.804 to 0.957)	0.468
Normalized (uHJV + uNGAL)	0.831 (0.751 to 0.893)	0.274
Normalized (uKIM-1 + uNGAL)	0.862 (0.760 to 0.932)	0.943
Normalized (uHJV + uKIM-1 + uNGAL)	0.897 (0.803 to 0.956)	0.496
Hour 6		
Normalized uHJV	0.808 (0.726 to 0.874)	NA
Normalized (uHJV + uKIM-1)	0.827 (0.719 to 0.906)	0.785
Normalized (uHJV + uNGAL)	0.808 (0.726 to 0.874)	1.00
Normalized (uKIM-1 + uNGAL)	0.816 (0.707 to 0.898)	0.712
Normalized (uHJV + uKIM-1 + uNGAL)	0.834 (0.728 to 0.912)	0.634
Hour 12		
Normalized uHJV	0.841 (0.715 to 0.927)	NA
Normalized (uHJV + uKIM-1)	0.890 (0.766 to 0.962)	0.332
Normalized (uHJV + uNGAL)	0.850 (0.725 to 0.933)	0.706
Normalized (uKIM-1 + uNGAL)	0.866 (0.736 to 0.947)	0.819
Normalized (uHJV + uKIM-1 + uNGAL)	0.892 (0.769 to 0.963)	0.311
Hour 24		
Normalized uKIM-1	0.772 (0.625 to 0.882)	NA
Normalized (uKIM-1 + uHJV)	0.848 (0.711 to 0.936)	0.137
Normalized (uKIM-1 + uNGAL)	0.826 (0.686 to 0.921)	0.230
Normalized (uHJV + uNGAL)	0.730 (0.587 to 0.844)	0.567
Normalized (uKIM-1 + uHJV + uNGAL)	0.855 (0.720 to 0.941)	0.085

*AUC* area under the receiver-operating characteristic curve, *CI* confidence interval, *NA* not applicable, *uHJV* urinary hemojuvelin, *uKIM-1* urinary kidney injury molecule-1, *uNGAL* urinary neutrophil gelatinase-associated lipocalin

^a^Hosmer-Lemeshow goodness of fit test: *p* = 0.541 for the best prediction model

^b^Compared with normalized uHJV at hour 3, hour 6 and hour 12 and normalized uKIM-1 at hour 24

^c^Best prediction model (greatest AUC)

### Adding urinary biomarkers to clinical risk prediction models

In combining the predictive ability of novel biomarkers and various clinical scoring systems for AKI, we found that the greatest AUC was observed with the combination of Liano’s AKI score and the panel of normalized uHJV and uKIM-1 at 3 h after surgery, with an AUC of 0.931 to predict advanced AKI. This panel had a greater AUC value than other combinations of biomarkers at 3, 6, 12, or 24 h after surgery (Table 4 and Fig. 2).

**Table 4:** Logistic regression analysis with variables available for predicting advanced acute kidney injury and model accuracy after combining clinical models with urinary biomarkers

Model	AUC (95% CI)	*p* ^b^
Liano’s score	0.619 (0.536 to 0.698)	
Cleveland score	0.493 (0.410 to 0.576)	
SOFA score	0.700 (0.619 to 0.773)	
Hour 3		
Normalized uHJV	0.833 (0.753 to 0.895)	NA
Liano’s + normalized uHJV	0.817 (0.736 to 0.882)	0.672
Cleveland + normalized uHJV	0.808 (0.726 to 0.874)	0.662
SOFA + normalized uHJV	0.786 (0.701 to 0.856)	0.419
Normalized (uHJV + uKIM-1)	0.898 (0.804 to 0.957)	0.468
Liano’s + normalized (uHJV + uKIM-1)^a,c^	0.931 (0.846 to 0.977)	0.206
Cleveland + normalized (uHJV + uKIM-1)	0.883 (0.785 to 0.946)	0.817
SOFA + normalized (uHJV + uKIM-1)	0.876 (0.776 to 0.942)	0.811
Hour 6		
Normalized uHJV	0.808 (0.726 to 0.874)	NA
Liano’s + normalized uHJV	0.791 (0.707 to 0.860)	0.653
Cleveland + normalized uHJV	0.769 (0.683 to 0.841)	0.544
SOFA + normalized uHJV	0.759 (0.672 to 0.833)	0.426
Normalized (uHJV + uKIM-1 + uNGAL)	0.834 (0.728 to 0.912)	0.634
Liano’s + normalized (uHJV + uKIM-1 + uNGAL)	0.867 (0.766 to 0.935)	0.334
Cleveland + normalized (uHJV + uKIM-1 + uNGAL)	0.817 (0.709 to 0.899)	0.985
SOFA + normalized (uHJV + uKIM-1 + uNGAL)	0.812 (0.702 to 0.895)	0.920
Hour 12		
Normalized uHJV	0.841 (0.715 to 0.927)	NA
Liano’s + normalized uHJV	0.834 (0.707 to 0.922)	0.879
Cleveland + normalized uHJV	0.892 (0.776 to 0.960)	0.429
SOFA + normalized uHJV	0.834 (0.705 to 0.923)	0.656
Normalized (uHJV + uKIM-1 + uNGAL)	0.892 (0.769 to 0.963)	0.311
Liano’s + normalized (uHJV + uKIM-1 + uNGAL)	0.923 (0.808 to 0.980)	0.151
Cleveland + normalized (uHJV + uKIM-1 + uNGAL)	0.916 (0.800 to 0.976)	0.281
SOFA + normalized (uHJV + uKIM-1 + uNGAL)	0.914 (0.795 to 0.975)	0.302
Hour 24		
Normalized uKIM-1	0.772 (0.625 to 0.882)	NA
Liano’s + normalized uKIM-1	0.799 (0.655 to 0.902)	0.488
Cleveland + normalized uKIM-1	0.765 (0.617 to 0.877)	0.737
SOFA + normalized uKIM-1	0.797 (0.650 to 0.902)	0.587
Normalized (uKIM-1 + uHJV + uNGAL)	0.855 (0.720 to 0.941)	0.085
Liano’s + normalized (uKIM-1 + uHJV + uNGAL)	0.882 (0.753 to 0.958)	0.030
Cleveland + normalized (uKIM-1 + uHJV + uNGAL)	0.863 (0.729 to 0.946)	0.096
SOFA + normalized (uKIM-1 + uHJV + uNGAL)	0.869 (0.735 to 0.951)	0.082

*AUC* area under the receiver-operating characteristic curve, *CI* confidence interval, *SOFA* score Sequential Organ Failure Assessment score, *uHJV* urinary hemojuvelin, *uKIM-1* urinary kidney injury molecule-1, *uNGAL* urinary neutrophil gelatinase-associated lipocalin

^a^Hosmer-Lemeshow goodness of fit test: *p* = 0.481 for the best prediction model

^b^Compared with normalized uHJV at hour 3, hour 6 and hour 12 and normalized uKIM-1 at hour 24

^c^Best prediction model (greatest AUC), combined clinical model (Liano’s score) with biomarkers

We assessed reclassification and evaluated any improvement in detection of advanced AKI events. The combination of the creatinine-normalized uHJV and uKIM-1 biomarker panel with the Liano’s score led to a significant increase in risk stratification (total NRI = 1.149; *p* < 0.001). The majority of this effect came from those with advanced AKI events (NRI event = 0.538; *p* < 0.001), whereas the NRI non-event was 0.610 (95% CI, *p* < 0.001) (NRI reclassification table and proportion was shown in Additional file 1: Table S2). Simultaneously, the total integrated discrimination improvement (IDI) was significant at 0.383 (95% CI, 0.25–0.52; *p* < 0.001). The combination of normalized uHJV and uKIM-1 with the Liano’s score was also significant for NRI and IDI reclassification in forecasting advanced AKI at 6, 12, and 24 h post-surgery (Table 5).

**Table 5:** Discriminative improvement of combined biomarkers added to Liano’s score for prediction of advanced acute kidney injury

Model	AUC (95% CI)	*p* ^a^	NRI^b^ (95% CI)	*p* ^c^	IDI (95% CI)	*p*
Hour 3						
Normalized (uHJV + uKIM-1)	0.898 (0.804 to 0.957)	NA				
Liano’s + normalized (uHJV + uKIM-1)^d^	0.931 (0.846 to 0.977)	0.330	1.149 (0.76 to 1.53)	< 0.001	0.383 (0.25 to 0.52)	< 0.001
Hour 6						
Normalized (uHJV + uKIM-1 + uNGAL)	0.834 (0.728 to 0.912)	NA				
Liano’s + normalized (uHJV + uKIM-1 + uNGAL)	0.867 (0.766 to 0.935)	0.499	1.030 (0.64 to 1.41)	< 0.001	0.343 (0.21 to 0.48)	< 0.001
Hour 12						
Normalized (uHJV + uKIM-1 + uNGAL)	0.892 (0.769 to 0.963)	NA				
Liano’s + normalized (uHJV + uKIM-1 + uNGAL)	0.923 (0.808 to 0.980)	0.344	0.831 (0.40 to 1.27)	< 0.001	0.353 (0.19 to 0.52)	< 0.001
Hour 24						
Normalized (uKIM-1 + uHJV + uNGAL)	0.855 (0.720 to 0.941)	NA				
Liano’s + normalized (uKIM-1 + uHJV + uNGAL)	0.882 (0.753 to 0.958)	0.288	1.162 (0.81 to 1.52)	< 0.001	0.387 (0.27 to 0.51)	< 0.001

*AUC* area under the receiver-operating characteristic curve, *CI* confidence interval, *IDI* integrated discrimination improvement, *NRI* net reclassification improvement, *uHJV* urinary hemojuvelin, *uKIM-1* urinary kidney injury molecule-1, *uNGAL* urinary neutrophil gelatinase-associated lipocalin

^a^Compared with normalized (uHJV + uKIM-1) at hour 3, normalized (uHJV + uKIM-1 + uNGAL) at hour 6, normalized (uHJV + uKIM-1 + uNGAL) at hour 12 and normalized (uKIM-1 + uHJV + uNGAL) at hour 24

^b^The ability of a risk marker to more accurately stratify individuals into higher or lower risk categories was investigated by NRI. We reclassified the patients who had subsequent advanced acute kidney injury (AKI) or who did not by using a priori risk categories of 0–12%, 12–30% and > 30% for the risk of advanced AKI

^c^The *p* value for increase in NRI in a model with urinary biomarkers combined with Liano’s score compared with urinary biomarkers alone

^d^Best prediction model (greatest AUC), combined clinical model (Liano’s score) with biomarkers

### Combining clinical risk prediction models and urinary biomarkers for prediction of mortality and composite outcomes

**Fig. 3:**
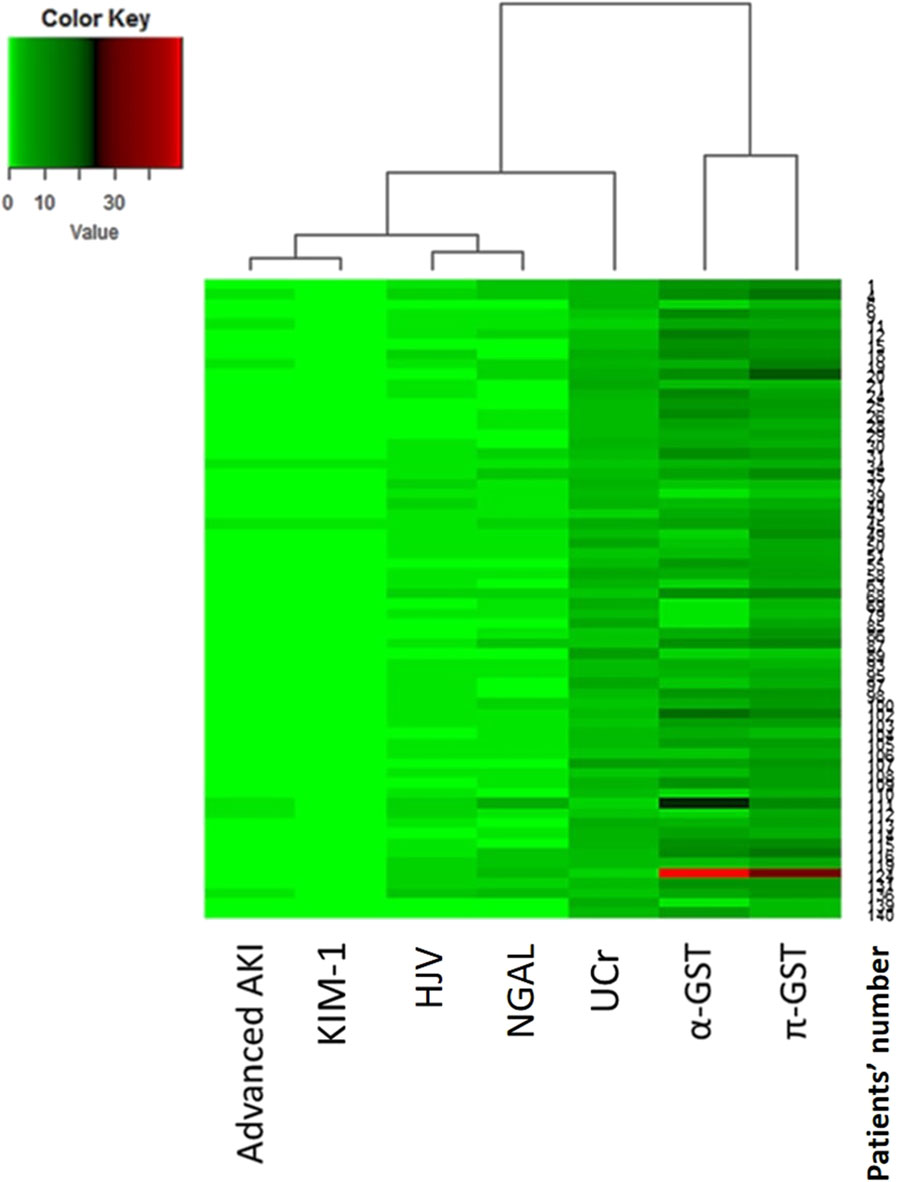
Urinary biomarker concentrations related to advanced acute kidney injury (AKI). The creatinine-normalized biomarker concentrations were analyzed by unsupervised clustering to determine their relationship to advanced AKI. Full-length view of the cluster diagram has cases orientated along the vertical axis and biomarkers orientated along the horizontal axis. α-GST, α-glutathione S-transferase; π-GST, π-glutathione S-transferase; HJV, hemojuvelin; KIM-1, kidney injury molecule-1; NGAL, neutrophil gelatinase associated lipocalin; UCr, urinary creatinine

The combined panel of normalized uHJV and uNGAL at 3 h post-surgery had strong predictive ability for 90-day mortality rate after hospital discharge (AUC = 0.896) and the best predictive ability for composite outcomes (AUC = 0.905). The predictive ability for composite outcomes further improved after combination with Liano’s score (AUC = 0.943) (Additional file 1: Table S3). Given the potential for collinearity between some of the biomarkers, we performed an unsupervised cluster analysis to determine the relationship of the biomarkers to advanced AKI (Fig. 3). The unsupervised clustering resulted in groupings similar to functional groupings for some of the biomarkers. For instance, uHJV and uKIM-1 in the group associated with proximal tubular dysfunction were clustered in the near group, nearest to “advanced AKI”, and may explain their strong predictive ability for advanced AKI. Overall, the correlation within each group was stronger for biomarkers that had better predictive ability.

## Discussion

In this prospective multicenter observational study, we have shown that the combined biomarker panel of creatinine-normalized uHJV and uKIM-1 at 3 h after cardiovascular surgery had the strongest predictive ability for the development of advanced AKI and composite outcomes. This predictive ability was increased further after combination with Liano’s score. The combination of clinical models and biologic biomarkers could better anticipate the future development of advanced AKI in the early postoperative period, suggesting future clinical outcomes.

The combination of uHJV and uKIM-1, both markers of structural kidney injury, provided added value to the traditional clinical AKI score in forecasting the risk of advanced AKI. Consequently, combination approaches could account for different time courses of biomarker release or could reflect different pathophysiological mechanisms [41]. HJV, a glycophosphatidylinositol (GPI)-linked membrane protein, is highly expressed in liver and skeletal muscles. The molecular weight of HJV is 42 kDa for the soluble form (sHJV) [42], and can be passed through glomerular filtration and reabsorbed by the renal tubules [8]. Increased iron content in the kidney and urine is observed in human and animal models of AKI [43], and increased iron load can induce renal tubular cell injury [44]. There is evidence that the increased expression of the hemojuvelin-hepcidin-ferroportin pathway is an intrinsic response to iron overload conditions during AKI. Therefore, uHJV has the potential to be an early AKI biomarker in response to iron homeostasis during AKI, which may explain the temporal relationship between uHJV and its predictive capacity. In this study, we showed that the novel biomarker of HJV can predict AKI after cardiac surgery. This finding reinforced our previous finding, in which high urinary HJV levels were observed in patients after cardiac surgery and rhabdomyolysis-related AKI. Although HJV is highly expressed in liver, the post-surgery liver function was not significantly different between the no AKI/stage 1 AKI and the stage 2/3 AKI groups This result suggested higher HJV concentration in patients with advanced AKI was mainly from kidney injury. According to our animal model, the elevation of urinary HJV in acute tubular necrosis should be originally from filtration after renal tubular destruction rather than the liver (see Additional file 1) [8].

Kidney injury molecule-1 (KIM-1), a 38.7-kDa transmembrane protein with immunoglobulin-like and mucin domains in its ectodomain, is a novel biomarker for renal proximal tubule injury and could play a role in tubulo-interstitial damage [45]. In addition to being a biomarker of AKI, KIM-1 could also play a role in renal recovery and tubular regeneration after AKI [46]. However, because increased urinary KIM-1 concentration can indicate either injury or the repair response to injury, KIM-1 by itself may not be a suitable marker for early prediction of AKI whereas the combination of KIM-1 with other injury markers may be highly useful. In support of this hypothesis, our result was consistent with a study investigating biomarker combinations for prediction of AKI following cardiac surgery [14], whereby KIM-1 in combination with another biomarker had the best predictive value for severe AKI.

We further showed that the combination of a panel of biomarkers and Liano’s AKI score may be beneficial in providing additional information with respect to the risk of AKI severity and other adverse clinical outcomes. Due to the heterogeneous causes of AKI and underlying comorbidities, the use of biomarkers and clinical disease severity scores to predict renal injury seems reasonable and actually did have better accuracy [17]. For increased accuracy and added predictive ability beyond the AUC value, the biomarker and clinical score combination model was supported by net reclassification improvement (NRI) and integrated discrimination improvement (IDI) values. The NRI and IDI represent modern, more sensitive statistical methods to reclassify model improvement from the use of a biomarker with an existing clinical model. Urinary HJV and uKIM-1 had the unique ability to improve the NRI for both events and non-events and augmented the AUC value compared with use of a clinical model alone and thus, uHJV and uKIM-1 may serve as an ideal biomarker panel to help early detection of advanced AKI.

Biomarkers predicting advanced AKI will allow future clinical trials in AKI intervention to be targeted to those patients at highest risk of disease progression, which would be far more efficient in terms of costs and resources [47]. The majority of patients (28/46, 60.9% in this study) who developed AKI after cardiovascular surgery had stage 1 AKI and this spontaneously resolved. The ability to distinguish those patients at highest risk of severe outcomes will be crucial as nephrologists and intensivists conduct more clinical trials in the treatment of AKI [48]. Given the clinical uncertainty and high complexity of medical decision-making around this issue (e.g., timing and duration of renal replacement therapy, placement of permanent access and follow up), any method that provides a modest improvement in clinical prediction could have clinical importance. We endeavor to find the biomarkers and clinical severity score to determine which patients will develop advanced AKI, and help to improve clinical decision-making - such as earlier or delayed dialysis [4, 49], guiding decision-making, patient counseling and facilitating enrollee selection in interventional trials of AKI.

There were several limitations to our study. First, because of the limited sample size, the statistical significance may be improved after recruiting more patient samples. Second, only those patients undergoing cardiovascular surgery were included in this study and consequently other serious causes of AKI, such as sepsis or drug-induced AKI, are not represented. The results of our study could add to the value of AKI prediction after generalized cardiovascular surgery, and further large prospective studies are necessary to validate our results. Third, about 3% of urinary biomarker data were lost due to anuria at 12 and 24 h post-surgery. In addition, we did not obtain urine samples during surgery. Tubular injury may occur 30 min after commencing cardiopulmonary bypass in cardiac surgery with a peak immediately following completion of cardiopulmonary bypass [50]. However, our results clearly showed these markers had their peak level at 3 h after surgery and could predict advanced AKI. For clinical practice, urine collection after surgery was more feasible than collection during surgery. Finally, other urinary biomarkers, such as IL-18 and L-fatty acid binding protein, which also have promising results for predicting AKI, were not examined [51, 52].

Our study also has several strengths. First, this was a large multicenter prospective study and included a homogenous (post-cardiovascular surgery) patient population. Second, our study contained five biomarkers (including markers of proximal and distal tubular injury) and tested urine samples obtained in the ICU at 0, 3, 6, 12 and 24 h after completion of surgery, which allowed exploration of the dynamic changes in their urinary concentrations and provided more comprehensive and credible results than previous studies [12, 53, 54]. Finally, this is, to our best knowledge, the first study using panels of urinary biomarkers in combination with clinical risk scores to focus on prediction of advanced AKI after cardiovascular surgery. Urinary biomarkers can lead to more accurate diagnosis of AKI and can further be used for recruitment of more homogenous patient populations when implementing a clinical trial [55].

## Conclusions

In conclusion, our results indicate that creatinine-normalized urinary biomarkers, particularly HJV and KIM-1 in combination, can improve the clinical predictive ability of Liano’s score for advanced AKI and composite outcomes at an early time point after cardiovascular surgery. Risk prediction utilizing both biomarkers and clinical AKI score may enhance critical care and aid in forecasting the prognosis of postoperative patients. Larger prospective studies are necessary to confirm our observations and to validate the predictive panel for assessing clinical AKI.

## Additional file

Additional file 1: Supplementary methods: brief introduction of HJV, KIM-1, uNGAL, α-GST, π-GST; Liano’s score, National Taiwan University Study Group on Acute Renal Failure (NSARF) introduction and inotropic equivalents (IE). **Table S1.** Area under the receiver-operating characteristic curve (AUC) for normalized uHJV, uKIM-1 and serum creatinine for predicting advanced AKI at 3 and 6 h post-surgery. **Table S2.** Number and percent reclassified in advanced AKI prediction comparing initial model (Normalized [uHJV + uKIM-1]) to updated model (Liano’s + Normalized [uHJV + uKIM-1]) at 3 h post-surgery. **Table S3.** Logistic regression analysis with variables available for predicting 90 days mortality after hospital discharge and composite outcomes. **Figure S1.** Receiver-operator characteristic curves for normalized uHJV, uKIM-1 and serum creatinine for predicting advanced AKI at 3 and 6 h post-surgery. **Figure S2.** Generalized additive models (GAM) plot for the probability of advanced AKI for normalized urinary HJV and KIM-1 at T3 (3 h post-surgery). (DOCX 723 kb)

## Abbreviations

AKI: Acute kidney injury
ANOVA: Analysis of variance
BMI: Body mass index
CABG: Coronary artery bypass graft
CCF ARF: Cleveland Clinic Foundation Acute Renal Failure
COPD: Chronic obstructive pulmonary disease
CPB: Cardiopulmonary bypass
eGFR: Estimated glomerular filtration rate
ELISA: Enzyme-linked immunosorbent assay
GAM: Generalized additive model
ICU: Intensive care unit
IDI: Integrated discrimination improvement
IL: Interleukin
kDa: KiloDalton
KDIGO: Kidney Disease: Improving Global Outcomes
MDRD: Modification of Diet in Renal Disease Study equation
NRI: Net reclassification improvement
SOFA: Sequential Organ Failure Assessment
uHJV: Urinary hemojuvelin
uKIM-1: Urinary kidney injury molecule-1
uNGAL: Urinary neutrophil gelatinase-associated lipocalin
uα-GST: Urinary α-glutathione S-transferase
uπ-GST: Urinary π-glutathione S-transferase
