# Supplementary material for: Urinary biomarkers predict advanced acute kidney injury after cardiovascular surgery
Source: Crit Care. 2018 Apr 26;22:108. doi: 10.1186/s13054-018-2035-8 (PMC5921971; doi:10.1186/s13054-018-2035-8)
Supplement: Supplementary file 1 — Supplementary methods: brief introduction of HJV, KIM-1, uNGAL, α-GST, π-GST; Liano’s score, National Taiwan University Study Group on Acute Renal Failure (NSARF) introduction and inotropic equivalents (IE). Table S1. Area under the receiver-operating characteristic curve (AUC) for normalized uHJV, uKIM-1 and serum creatinine for predicting advanced AKI at 3 and 6 h post-surgery. Table S2. Number and percent reclassified in advanced AKI prediction comparing initial model (Normalized [uHJV + uKIM-1]) to updated model (Liano’s + Normalized [uHJV + uKIM-1]) at 3 h post-surgery. Table S3. Logistic regression analysis with variables available for predicting 90 days mortality after hospital discharge and composite outcomes. Figure S1. Receiver-operator characteristic curves for normalized uHJV, uKIM-1 and serum creatinine for predicting advanced AKI at 3 and 6 h post-surgery. Figure S2. Generalized additive models (GAM) plot for the probability of advanced AKI for normalized urinary HJV and KIM-1 at T3 (3 h post-surgery). (DOCX 723 kb) [file 13054_2018_2035_MOESM1_ESM.docx]

**Supplementary Material**

**Brief introduction of HJV, KIM-1, uNGAL, α-GST and, π-GST**

HJV: The molecular weight of soluble form hemojuvelin (sHJV) is 42 kDa [[1](file:///C:\Users\JianJhong%20Wang\Desktop\奇美醫院\NSARF\Study\Biomarkers\正式Paper\投稿\CC\文章\Supplementary%20Material%20(R2)%20(for%20Rory)_reviewed.docx#_ENREF_1)], can be passed through glomerular filtration and reabsorbed by renal tubules. In humans, urinary HJV could not be identified from patients with normal kidney function even in nephrotic syndrome patients with heavy proteinuria. However, urinary HJV increased after kidney injury, with HJV prominently stained in the proximal renal tubules after hypovolemic shock-related AKI [[2](file:///C:\Users\JianJhong%20Wang\Desktop\奇美醫院\NSARF\Study\Biomarkers\正式Paper\投稿\CC\文章\Supplementary%20Material%20(R2)%20(for%20Rory)_reviewed.docx#_ENREF_2)]. HJV was highly expressed in liver, skeletal muscle and heart [[1](file:///C:\Users\JianJhong%20Wang\Desktop\奇美醫院\NSARF\Study\Biomarkers\正式Paper\投稿\CC\文章\Supplementary%20Material%20(R2)%20(for%20Rory)_reviewed.docx#_ENREF_1)], however, at acute tubular necrosis, the elevation of urinary sHJV should be originally from filtration after renal tubular destruction [[2](#_ENREF_2)].

In our further animal study, HJV was injected into mice and exogenous HJV was determined by ELISA from both plasma and urine at 3, 6, 9 hours after injection. C57BL/6 mice were injected with 10 µg rmHJV protein, or PBS as a control. Before index time, the mice received an intravenous injection of a volume of 100 µl PBS with 10 µg rmHJV protein (R&D Systems) or PBS only. Mice plasma and urine HJV was measured using an ELISA kit (Quantikine® ELISA Mouse/Rat RGM-C/Hemojuvelin Immunoassay, with a standard measuring range of 31.3 - 2,000 pg/mL and the mean minimum detectable dose was 2.25 pg/mL). (supplementary figure)

The result indicates the secretion of sHJV from kidney is limited even with high plasma levels of sHJV under physiological kidney conditions, p<0.001.

KIM-1: kidney injury molecule-1 (KIM-1) is a 38.7-kDa transmembrane protein that contains extracellular mucin and Ig domains [[3](file:///C:\Users\JianJhong%20Wang\Desktop\奇美醫院\NSARF\Study\Biomarkers\正式Paper\投稿\CC\文章\Supplementary%20Material%20(R2)%20(for%20Rory)_reviewed.docx#_ENREF_3)]. Kidney injury molecule 1 (KIM-1) is highly overexpressed in dedifferentiated proximal tubule cells after ischemic or nephrotoxic AKI in animal models. It is expressed in proximal tubule cells and is thought to promote apoptotic and necrotic cell clearance. Upon injury, KIM-1 is upregulated and released into the urine and extracellular space and does not pass through glomerular filtration [[4](#_ENREF_4)].

NGAL: Human neutrophil gelatinase-associated lipocalin (NGAL) was originally identified as a 25 kDa protein covalently bound to gelatinase from neutrophils. It is produced by neutrophils and is expressed to a limited degree in the liver, spleen and kidney. NGAL is filtered through the glomerulus and taken up by the proximal tubule through megalin [[5](file:///C:\Users\JianJhong%20Wang\Desktop\奇美醫院\NSARF\Study\Biomarkers\正式Paper\投稿\CC\文章\Supplementary%20Material%20(R2)%20(for%20Rory)_reviewed.docx#_ENREF_5)]. It is the established inflammatory biomarker identified both in proximal and distal renal tubular damage [[6](#_ENREF_6)]

α-GST and π-GST:  The molecular weight of GST is about 25 kDa. As a constitutive cytoplasmatic enzyme, GST is enriched in the renal tubular epithelial cells and is detectable in the urine when the cell integrity of renal tubules is damaged. Immunohistochemistry examinations demonstrated the localization of α - and π -GST in the proximal and distal renal tubules, respectively [[7](#_ENREF_7)].

**Liano’s score**

The Liano’s score was generated from 328 patients with acute renal failure admitted to a single center from November 1977 to June 1988. Liano’ score contained several risk factors that are frequently used in other AKI scoring systems (mechanical ventilation, age, gender, hypotension, liver failure, oliguria, consciousness disturbance) ^[^[^8^](#_ENREF_8)^]^, and used for mortality prediction after AKI.

Liano’s score [[8](#_ENREF_8)] : probability of death = (0.032 . age decades) – (0.086 . male) – (0.109 . nephrotoxic) + (0.109 . oliguria) + (0.116 . hypotension) + (0.122 . jaundice) + (0.150 . coma) – (0.154 . consciousness) + (0.182 . assisted respiration) + 0.210.

**NSARF (National Taiwan University Study Group on Acute Renal Failure)**

National Taiwan University Study Group on Acute Renal Failure (NSARF) was initially founded by a party of nephrologists from the National Taiwan University hospital in 2004. The first work of NSARF was to prospectively establish a database of patients undergoing renal replacement therapy. To assure the quality and completeness of this data, NSARF picked patients from the intensive care units (ICUs) in the tertiary hospital (National Taiwan University Hospital) and its branch hospitals in Taiwan. All clinical information was recorded on a predetermined spreadsheet with regular check-ups by principle investigators to ensure data quality. Using the database, the NSARF investigators have conducted many studies with detailed results.

Today, the NSARF consists of a multidisciplinary group of physicians, statisticians, administrative managers and laboratory operators working across one medical center (National Taiwan University Hospital (NTUH), Taipei, Taiwan), and five regional hospitals (Cardinal Tien Hospital, New Taipei City, Taiwan; Taipei Tzu Chi Hospital, New Taipei City, Taiwan; Yun- Lin Branch of NTUH, Douliou City, Taiwan; Hsin-Chu Branch of NTUH, Hsin-Chu City, Taiwan; Chi-Mei Medical Center, Liouying, Tainan City, Taiwan).

**Inotropic equivalents (IE)**

Inotropic equivalents (IE, µg/kg/min) = dopamine + dobutamine + 100 X epinephrine + 100 X NE + 100 X isoproterenol + 15 X milrinone

**Table S1 Area under the receiver-operating characteristic curve (AUC) for normalized uHJV, uKIM-1 and serum creatinine for predicting advanced AKI at 3 and 6 hours post-surgery.**

| Advanced AKI | T3, AUC (95% CI) | T6, AUC (95% CI) |
| --- | --- | --- |
| Normalized uHJV | 0.833 (0.753 to 0.895) | 0.808 (0.726 to 0.874) |
| Serum Cr | 0.546 (0.462 to 0.627) | 0.668 (0.564 to 0.761) |
| AUC comparison | p = 0.001 | p = 0.282 |
| Advanced AKI | T3, AUC (95% CI) | T6, AUC (95% CI) |
| Normalized uKIM-1 | 0.819 (0.710 to 0.900) | 0.787 (0.675 to 0.875) |
| Serum Cr | 0.546 (0.462 to 0.627) | 0.668 (0.564 to 0.761) |
| AUC comparison | p = 0.037 | p = 0.349 |

*AKI* area under the receiver-operating characteristic curve, *CI* confidence interval, *uHJV* urinary hemojuvelin, *uKIM-1* urinary kidney injury molecule-1

**Table S2 Number and percent reclassified in advanced AKI prediction comparing initial model (Normalized [uHJV + uKIM-1]) to updated model (Liano’s + Normalized [uHJV + uKIM-1]) at 3 hours post-operation.**

*AKI* area under the receiver-operating characteristic curve, *CI* confidence interval, *NRI* Net reclassification indices, *uHJV* urinary hemojuvelin, *uKIM-1* urinary kidney injury molecule-1

**Table S3 Logistic regression analysis with variables available for predicting 90 days mortality after hospital discharge and composite outcomes.**

| Model^a^ | Mortality | | Composite outcomes^b^ | |
| --- | --- | --- | --- | --- |
|  | AUC (95% CI) | P^c^ | AUC (95% CI) | P^c^ |
| **Hour 3** |  |  |  |  |
| Normalized uHJV | 0.905 (0.838 to 0.951) | NA | 0.867 (0.793 to 0.922) | NA |
| Normalized uKIM-1 | 0.810 (0.700 to 0.893) | 0.337 | 0.834 (0.728 to 0.911) | 0.379 |
| Normalized uNGAL | 0.719 (0.629 to 0.798) | 0.007 | 0.696 (0.605 to 0.777) | 0.003 |
| **Normalized [uHJV + uKIM-1]** | **0.879 (0.781 to 0.944)** | **0.425** | **0.905 (0.813 to 0.961)** | **0.650** |
| Normalized [uHJV + uNGAL] | 0.896 (0.827 to 0.945) | 0.741 | 0.862 (0.787 to 0.919) | 0.681 |
| Normalized [uKIM-1 + uNGAL] | 0.871 (0.771 to 0.938) | 0.896 | 0.883 (0.785 to 0.946) | 0.851 |
| **Liano’s + Normalized [uHJV + uKIM-1]** | **0.890 (0.794 to 0.952)** | **0.830** | **0.943 (0.862 to 0.984)** | **0.216** |
| Liano’s + Normalized [uHJV + uNGAL] | 0.889 (0.818 to 0.939) | 0.742 | 0.855 (0.779 to 0.913) | 0.712 |
| Liano’s + Normalized [uKIM-1 + uNGAL] | 0.886 (0.789 to 0.949) | 0.922 | 0.913 (0.822 to 0.966) | 0.742 |

^a^Urinary biomarker at 3 hours after cardiovascular surgery.

^b^Composite outcomes is defined as mortality or dialysis dependence.

^c^Compared with normalized uHJV at 3 hours after cardiovascular surgery.

*AUC* area under the receiver-operating characteristic curve, *CI* confidence interval, *uHJV* urinary Hemojuvelin, *uKIM-1* urinary Kidney Injury Molecule-1, *uNGAL* urinary neutrophil gelatinase-associated lipocalin

**Figure. S1**

**A**


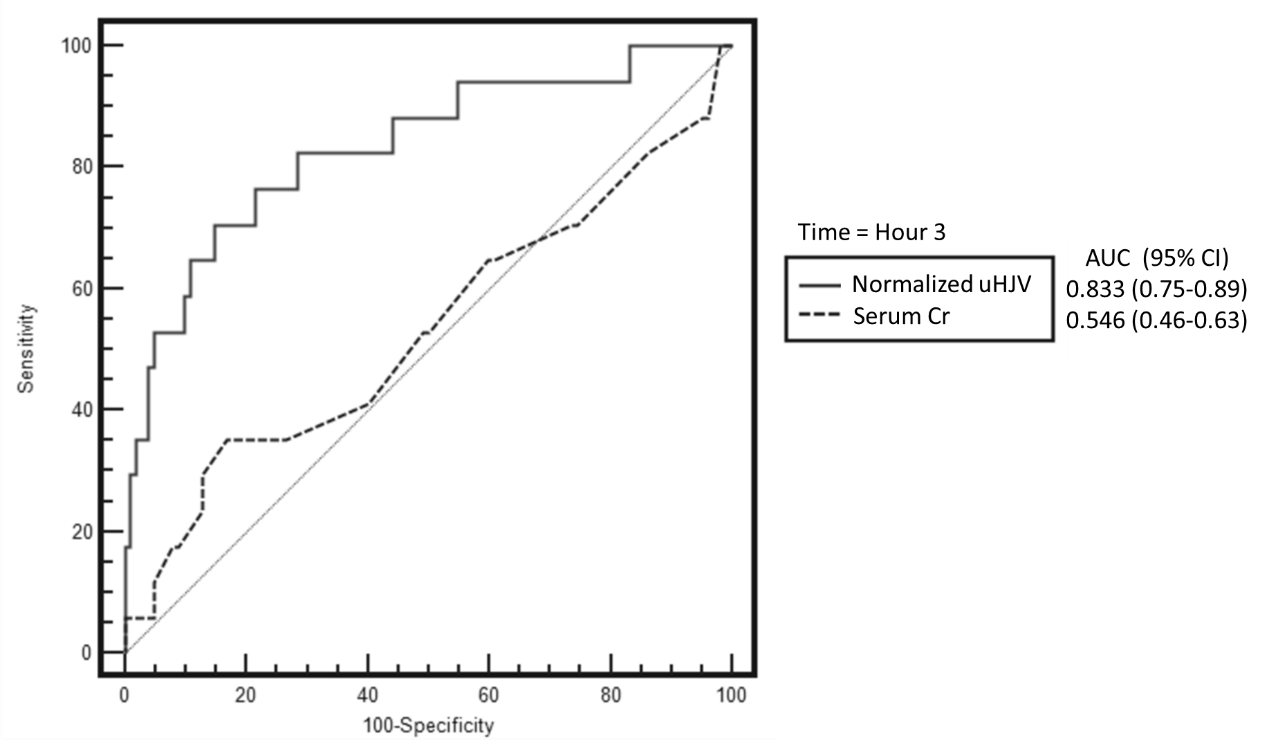


**B**


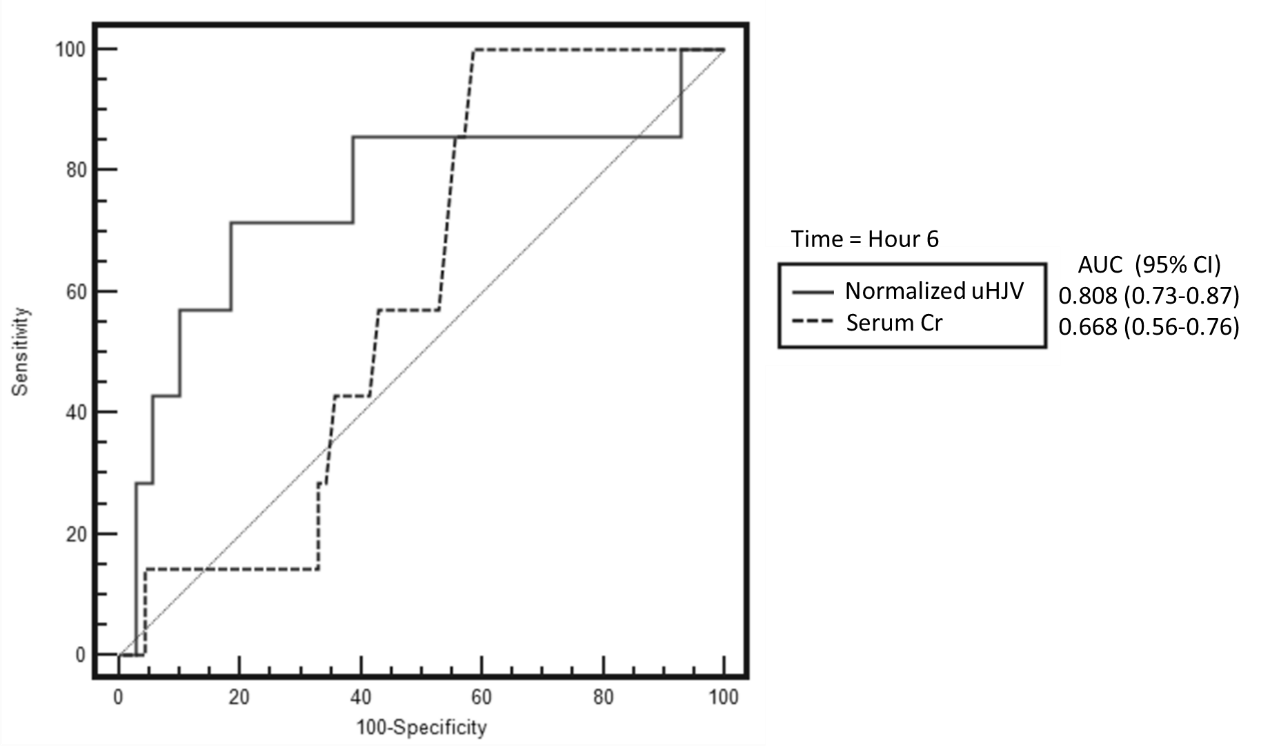


**C**


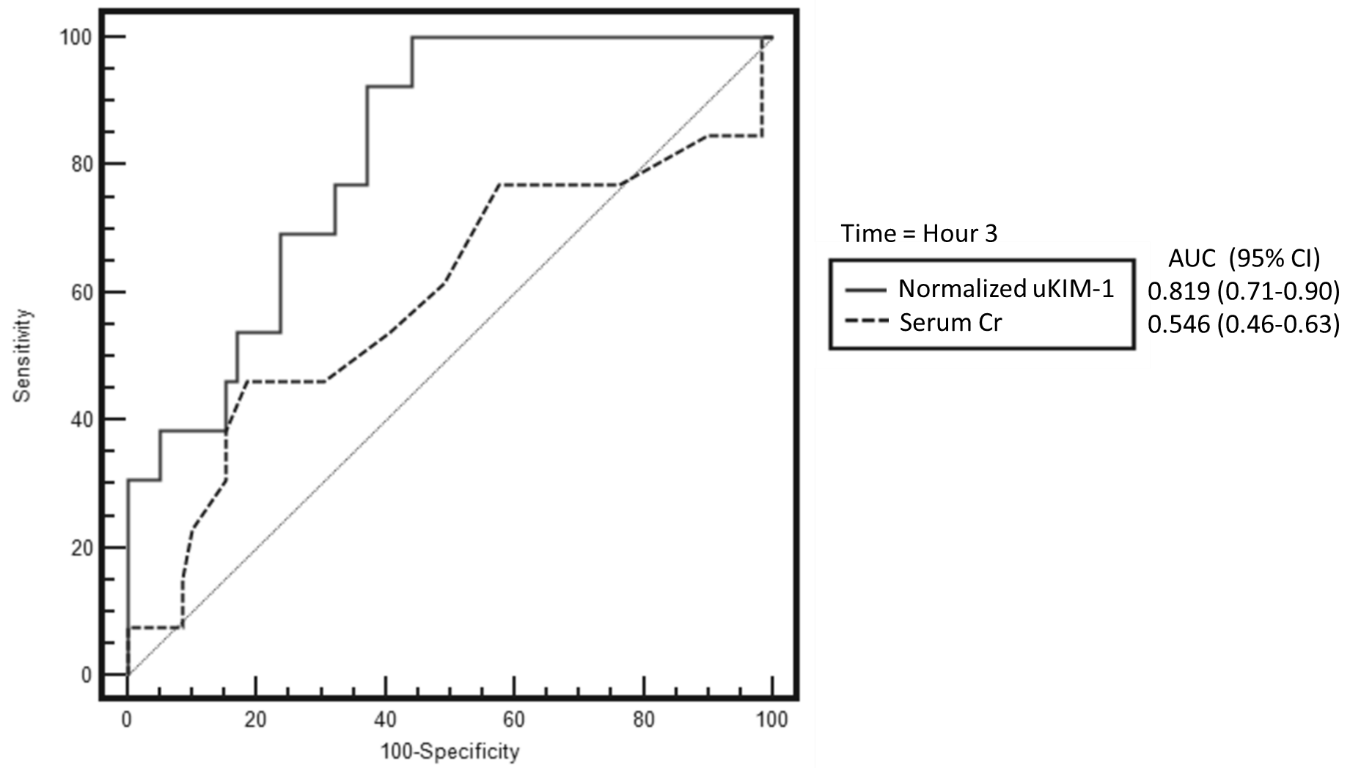


**D**


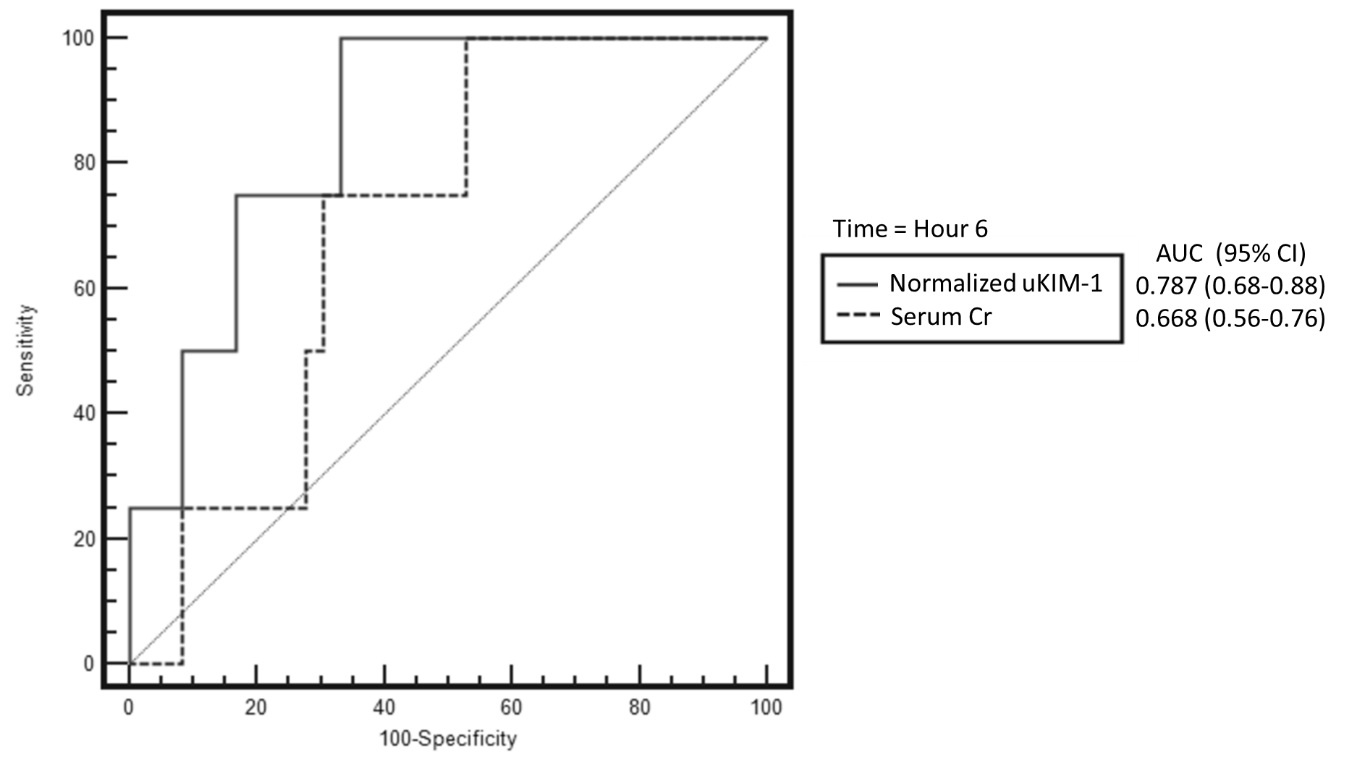


**Figure. S1 Receiver-operator characteristic curves for normalized uHJV, uKIM-1 and serum creatinine for predicting advanced AKI at 3 and 6 hours post-surgery.**

Normalized uHJV and serum creatinine at 3 hours (A) and 6 hours (B) post-surgery. Normalized uKIM-1 and serum creatinine at 3 hours (C) and 6 hours (D) post-surgery. The area under the ROC curve (AUC) values and 95% confidence intervals (CIs) are also shown.

**Figure. S2**

**A**


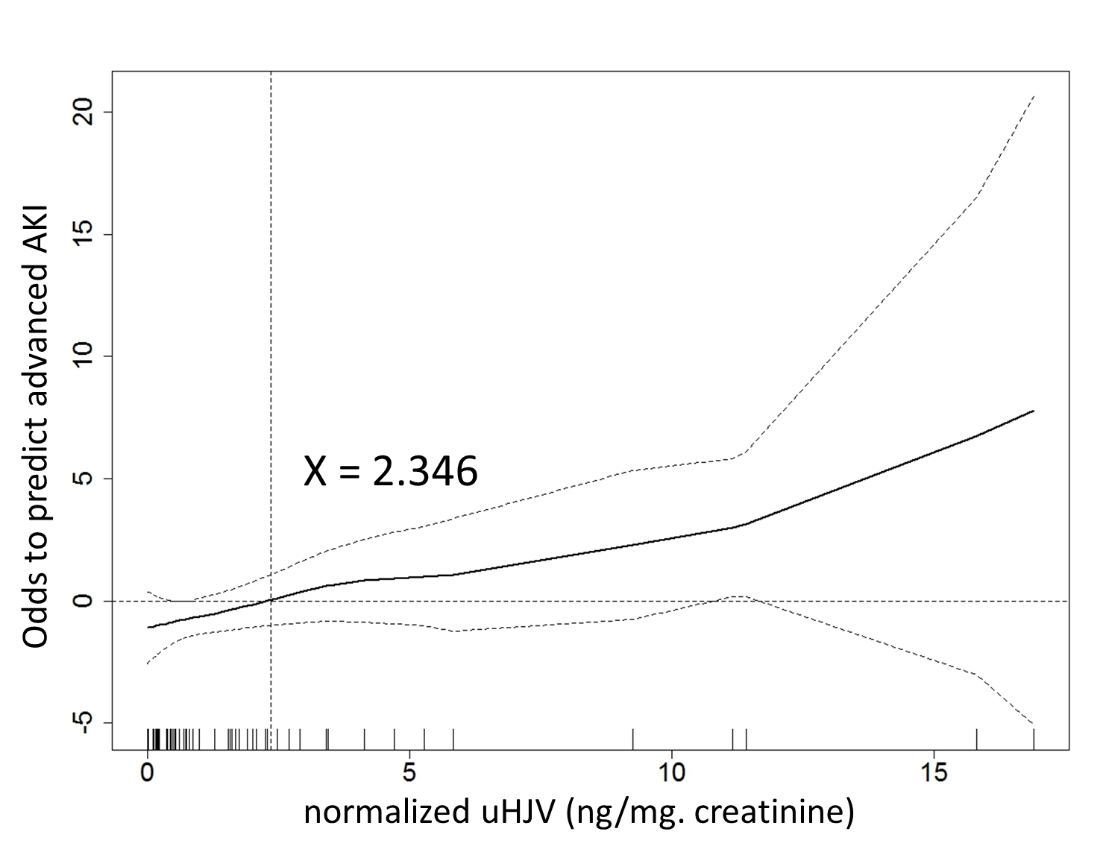


**B**


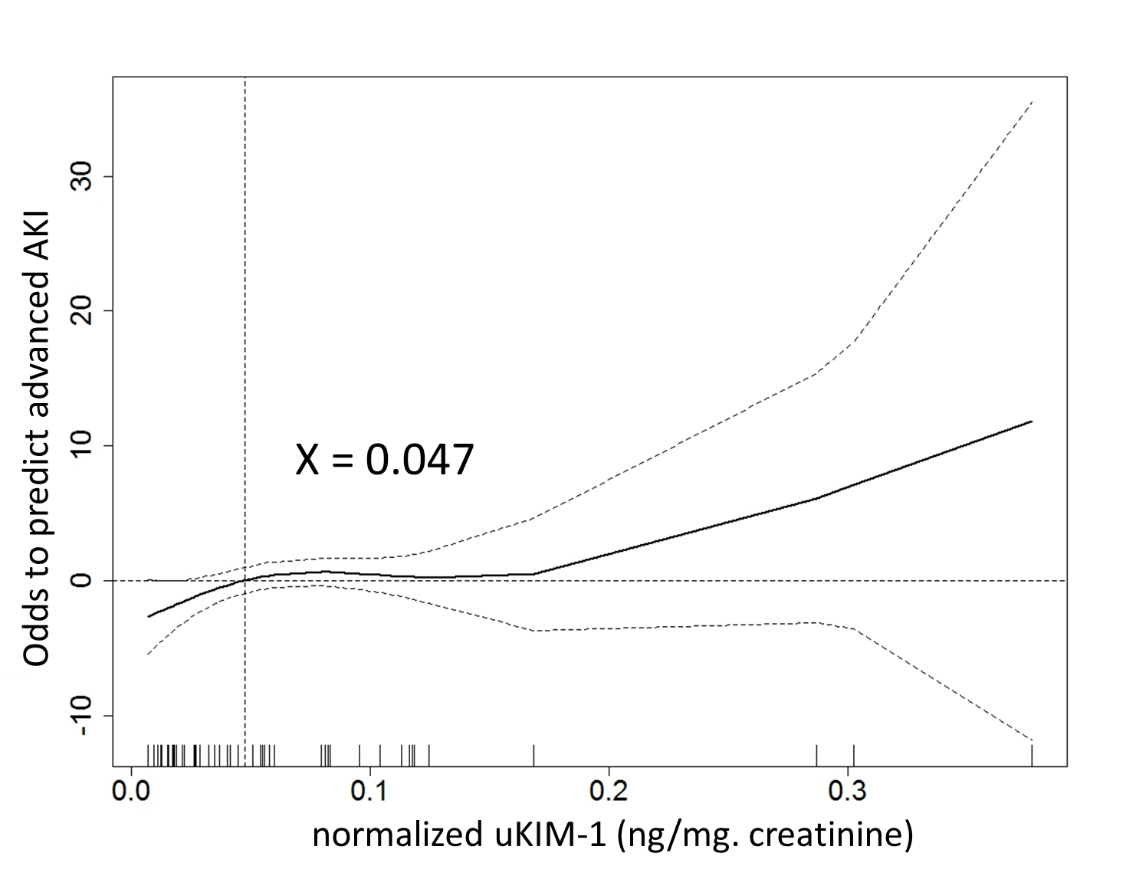


**Figure S2 Generalized additive model (GAM) plots for predicting the probability of advanced AKI using normalized urinary HJV and KIM-1 at T3 (3 hours post surgery).**

The model incorporates the subject-specific (longitudinal) random effects. The probability of outcome events was constructed with HJV (A) and KIM-1 (B) level and was centered to have an average of zero over the range of the data as constructed with the GAM. The cutoff value of 2.346 ng/mL for normalized uHJV and 0.047 ng/mL for normalized uKIM-1 showed the best performance to predict advanced AKI.

Reference

1. Lin L, Goldberg YP, Ganz T: **Competitive regulation of hepcidin mRNA by soluble and cell-associated hemojuvelin**. *Blood* 2005, **106**(8):2884-2889.

2. Young GH, Huang TM, Wu CH, Lai CF, Hou CC, Peng KY, Liang CJ, Lin SL, Chang SC, Tsai PR *et al*: **Hemojuvelin modulates iron stress during acute kidney injury: improved by furin inhibitor**. *Antioxidants & redox signaling* 2014, **20**(8):1181-1194.

3. Alge JL, Arthur JM: **Biomarkers of AKI: a review of mechanistic relevance and potential therapeutic implications**. *Clinical journal of the American Society of Nephrology : CJASN* 2015, **10**(1):147-155.

4. Charlton JR, Portilla D, Okusa MD: **A basic science view of acute kidney injury biomarkers**. *Nephrology, dialysis, transplantation : official publication of the European Dialysis and Transplant Association - European Renal Association* 2014, **29**(7):1301-1311.

5. Nguyen MT, Devarajan P: **Biomarkers for the early detection of acute kidney injury**. *Pediatric nephrology (Berlin, Germany)* 2008, **23**(12):2151-2157.

6. Srisawat N, Wen X, Lee M, Kong L, Elder M, Carter M, Unruh M, Finkel K, Vijayan A, Ramkumar M *et al*: **Urinary biomarkers and renal recovery in critically ill patients with renal support**. *Clinical journal of the American Society of Nephrology : CJASN* 2011, **6**(8):1815-1823.

7. Harrison DJ, Kharbanda R, Cunningham DS, McLellan LI, Hayes JD: **Distribution of glutathione S-transferase isoenzymes in human kidney: basis for possible markers of renal injury**. *Journal of clinical pathology* 1989, **42**(6):624-628.

8. Liano F, Gallego A, Pascual J, Garcia-Martin F, Teruel JL, Marcen R, Orofino L, Orte L, Rivera M, Gallego N *et al*: **Prognosis of acute tubular necrosis: an extended prospectively contrasted study**. *Nephron* 1993, **63**(1):21-31.
